# Frailty as a determinant of mortality, surgical timing and hospital stay in proximal femur fractures: a retrospective cohort study

**DOI:** 10.1007/s00590-025-04312-6

**Published:** 2025-05-14

**Authors:** Rocco Maria Comodo, Emidio Di Gialleonardo, Guido Bocchino, Giacomo Capece, Marcello Covino, Benedetta Simeoni, Andrea Russo, Sara Salini, Giulio Maccauro, Raffaele Vitiello

**Affiliations:** Catholic University of the Sacred Heart, Milan, Italy

**Keywords:** Frailty, Proximal femur fractures, Clinical frailty scale (CFS), Surgical timing, Hospital length of stay (LOS)

## Abstract

**Background:**

Proximal femur fractures are common in elderly patients and are strongly associated with frailty, increased mortality, and functional decline. The Clinical Frailty Scale (CFS) has emerged as a key predictor of outcomes, influencing perioperative management and rehabilitation strategies. This study aims to evaluate the impact of frailty on mortality, surgical timing, and length of hospital stay (LOS) in patients with hip fractures.

**Methods:**

This single-center retrospective study analyzed data from patients aged ≥ 65 years admitted with AO type 31A and 31B proximal femur fractures between 2018 and 2023. Patients were stratified according to CFS scores to assess the relationship between frailty and in-hospital mortality, surgical delay, and LOS. Multivariate logistic regression was performed to identify independent risk factors.

**Results:**

Among 2312 patients (median age: 85 years), frailty was a significant predictor of mortality (*p* = 0.019). Patients with CFS 7–9 had a 6.6-fold higher mortality risk than those with CFS 1–3. Delays in surgery beyond 48 h were associated with a doubled risk of mortality (*p* = 0.009), particularly in frail patients. Prolonged LOS (> 7.1 days) correlated with an increased incidence of infections, cardiovascular events, and mortality (*p* < 0.001).

**Conclusions:**

Frailty significantly impacts hip fracture prognosis in elderly patients, increasing mortality, complications, and LOS. Early identification of frail patients and prioritization of timely surgery are crucial to improving outcomes. Future studies should explore tailored surgical strategies and optimized rehabilitation protocols to enhance recovery in this high-risk population.

## Introduction

Proximal femur fractures predominantly affect elderly patients and are associated with high mortality and disability rates [1]. These fractures, in elderly patients, are closely linked to osteoporosis, representing a pattern of fragility fractures [2]. Several factors may influence bone quality and the prevention of fragility fractures, including a healthy diet and adequate physical activity, whereas smoking and excessive alcohol consumption reduce bone mineral density (BMD), predisposing individuals to fractures [3]. Falling is a major cause of hip fractures in elderly patients. It has been estimated that 30% of individuals over 65 years and 50% of those over 80 experience at least one fall per year, with approximately 1% of cases resulting in a hip fracture [4]. The biomechanics of falls play a crucial role in determining the risk of femoral fractures. Recent studies have demonstrated that lateral falls increase the risk of fracture more than fivefold, while direct impact on the hip raises the risk more than threefold [5]. Women seem to be more affected than men; however, in male patients, the one-year mortality rate following a hip fracture is higher [6]. With the increasing average age of the population, the annual incidence of proximal femur fractures is projected to reach between 6.3 million and 8.2 million cases by 2050 [7]. The economic burden of hip fractures is substantial, primarily due to hospitalization and rehabilitation costs [6].

Mortality rates following proximal femur fractures vary depending on several factors. The literature shows that the major determinants are the site of the fracture, the patient’s fragility status, the waiting time for surgery, and the duration of hospitalisation.

Frailty is defined as a vulnerable state resulting from diminished functional reserves due to aging [8]. Upon admission to the emergency department (ED), hip fracture patients often present with frailty and multiple comorbidities. The assessment of frailty status is crucial for stratifying the risk of in-hospital mortality [9]. A study by Narula et al. [10] highlighted that the Clinical Frailty Score (CFS) is an effective predictor of both 30-day and one-year mortality in patients with proximal femur fractures.

Therefore, preoperative assessment is crucial for identifying risk factors that influence mortality, such as pulmonary disease, cardiovascular disease, diabetes, or nursing home residence [11]. Several clinical scoring systems, in addition to the previously mentioned CFS, are employed to stratify mortality risk in hip fracture patients. The Nottingham Hip Fracture Score (NHFS) is frequently used to predict 30-day mortality and is also linked to hospital length of stay and post-surgical functional limitations [12]. Furthermore, the American Society of Anesthesiologists (ASA) score and the Charlson Comorbidity Index (CCI) are established models for predicting one-year mortality in hip fracture patients. A study by Ek et al. [13] showed that both the CCI and ASA score are effective tools for assessing mortality risk.

The aim of our study is to evaluate the influence of frailty status in patients with hip fractures upon admission to the emergency department, in particular how different CFS scores relate to patient mortality and adverse outcomes. Similarly, our study seeks to highlight how a patient’s frail condition complicates their management, leading to an increased length of stay (LOS). LOS is closely correlated with mortality and the onset of complications, underscoring the need to recognize and manage frail patients early upon admission to the emergency department.

Finally, by analyzing the profiles of deceased patients, we will attempt to retrospectively understand whether there are modifiable or non-modifiable risk factors that may increase the risk of death. Therefore, we aim to identify these factors upon admission to the emergency department and treat them promptly.

## Methods

### Study population and design

This single-center retrospective study was conducted at our hospital’s emergency department between January 2018 and October 2023. The study adhered to the principles outlined in the Declaration of Helsinki and its subsequent amendments and received approval from the Institutional Review Board of Fondazione Policlinico Universitario “A Gemelli” IRCCS – Rome (IRB #005181419). Informed consent was obtained from all participants prior to their inclusion in the analysis.

The study population comprised patients aged ≥ 65 years diagnosed with proximal femur fractures classified as types 31A and 31B according to the AO classification system [14] with a surgical indication.

Patients aged < 65 years were excluded from the study. Additionally, exclusion criteria included patients with:

(I) Isolated greater trochanteric fractures, acetabular fractures, and pelvic fractures,
(II) Pathological fractures,
(III) Periprosthetic hip fractures,
(IV) Femoral head fractures (31C),
(V) Refusal of surgical treatment.

Furthermore, patients who declined hospital admission were also excluded.

### Study variables

All patients were assessed in the ED to retrieve the following clinical and demographic data:

- Age, gender; type of fracture (31A or 31B)
- Overall frailty was assessed by the Clinical Frailty Scale (CSF). According to this scale, the patients were further categorized as “non-frail” for CSF 1–3 (corresponding to fit and mild vulnerability), “mild frail” for CSF 4–6 (corresponding to vulnerable or mild frail), and “frail” for CSF 7–9 (corresponding to moderate to severely frail). The frailty was assessed by a dedicated team (“frailty unit”), in the first 24 h since admission.
- The following laboratory values were assessed for each patient: Hemoglobin (Hb), blood glucose, fibrinogen, prothrombin time (PT),white blood cells (WBC), platelets (PLT). The National Early Warning Score (NEWS) score and Nottingham hip fracture score (NHFS) were calculated for each patient.
- Clinical history and comorbidities: the overall presence of comorbidities was evaluated using the Charlson Comorbidity Index for each patient. Comorbidities were determined based on patient reports and available medical records.
- The waiting time before surgery and the length of stay were calculated for each patient. Finally, the occurrence of any post-operative complications or in-hospital deaths among the patients was reported.

### Study endpoints

The primary study endpoint was to evaluate the influence of frailty status in patients with hip fractures upon admission to the emergency department, specifically how different CFS scores correlate with patient in-hospital mortality or adverse outcomes. 

### Statistical analysis

#### Multivariate analysis was performed

Continuous variables were reported as median [interquartile range], and are compared at univariate analysis by Mann–Whitney U test or Kruskal–Wallis test in case of three or more groups. Categorical variables were reported as absolute number (percentage) and are compared by Chi- square test (with Fisher’s test if appropriate).

Length of hospital stay was calculated from the time of ED admission to discharge or death. Survival curves were estimated by the Kaplan–Meier methods.

The study variables were assessed for the association to all-cause in-hospital death by a univariate Cox regression analysis. The variables reaching statistical significance at the univariate analysis were entered into a multivariate Cox regression model to identify independent risk factors for survival. We categorized all the continuous variables into dichotomous parameters (i.e. low/high) for a better model fitting. For each variable, we obtained the optimal dividing cut-off by Youden’s index, performing a Receiver operating characteristic (ROC) curve analysis for the association with death. Multivariate models excluded the single items composing any derived variable, both to avoid model overfitting and parameters overestimation. For this reason, all multivariate models included shock and excluded single physiological parameters and the NEWS. Similarly, the analysis considered a sum of comorbidities ≥ 3 and excluded both the single comorbidities and the CCI. The risk of intra-hospital death and major complications was expressed as Hazard Ratio (HR) [95% confidence interval]. A two-sided p ≤ 0.05 was considered significant in all the analyses. Data were analyzed by SPSS v25® (IBM, Armonk NY, USA) and MedCalc v18® (MedCalc Software Ltd, Ostend, Belgium).

## Results

According to inclusion and exclusion criteria, 2312 patients were considered eligible and included in the study. The median age of the patients was 85 years (IQR: 79–90), with a predominant distribution of female patients (75.2%) compared to males (24.8%).

Through multivariate logistic regression, CFS was identified as a significant determinant of in-hospital mortality (p = 0.019). Specifically, when compared to patients with mild CFS (1–3), those with moderate CFS (4–6) exhibited a 4.35-fold increased risk of death (OR = 4.351, p = 0.044), while patients with severe CFS (7–9) faced a 6.6-fold higher risk (OR = 6.609, p = 0.012). Age was also confirmed as another independent risk factor: for each additional year of age, the mortality risk rose by 6.5% (OR = 1.065, p < 0.001). Male sex correlated with a poorer prognosis, showing a 1.82-fold increase in mortality risk compared to female sex (OR = 1.824, p = 0.007). Regarding comorbidities, the Charlson Comorbidity Index, which measures the burden of pre-existing conditions, revealed a significant association with mortality: for each additional point in the Charlson score, the risk of death increased by 28.3% (OR = 1.283, p < 0.001). The multivariate analysis did not find a statistically significant association between fracture type and mortality (p = 0.827). However, among the various fracture types, subtrochanteric fractures exhibited a slightly higher, albeit non-significant, risk (OR = 1.072, p = 0.846). A critical finding was the waiting time for surgery, confirmed as a strong predictor of mortality (p = 0.020).

Patients who were operated on between 24 and 48 h after hospital admission faced a 1.47 times higher risk of death compared to those operated on within 24 h (OR = 1.468, p = 0.088), whereas patients waiting longer than 48 h showed a risk of mortality that was more than doubled (OR = 2.505, p = 0.009). Additionally, deceased patients had higher blood glucose levels (137 mg/dL vs. 124 mg/dL, p = 0.001) and fibrinogen levels (395.5 mg/dL vs. 353 mg/dL, p = 0.001) (Table 1).

**Table 1:** Clinical characteristics of enrolled patients according to survivorship (CCI = Charlson Comorbidity Index; CFS = Clinical Frailty Scale; ER = Emergency Room; LOS = Length of Stay; Hb = Hemoglobin; COPD = Chronic Obstructive Pulmonary Disease; Q1–Q3 = Interquartile range (25th–75th percentile)

Variable	Survived patients (Q1–Q3) (n = 2214)	Deceased patients (Q1–Q3) (n = 98)
Age (years)	84 (78–90)	88 (84–92)
CCI	4 (4–5)	5 (4–6)
CFS adjusted	5 (4–6)	6 (4–7)
Nottingham mortality (%)	2,371 (1,451–3,849)	3,849 (2,371–6,192)
ER stay (hours)	20.1 (9.8–28.6)	24.9 (13.775–39.775)
LOS (days)	7.075 (5–11.211)	12.21 (4.982–21.955)
Time to surgery (hours)	20.6 (10.2–29.425)	25.4 (17.15–41.625)
*Length of stay (LOS)*		
LOS < 7.1 days (%)	1123 (50.7)	33 (33.7)
LOS > 7.1 days (%)	1091 (49.3)	65 (66.3)
*Laboratory values*		
Hb (g/dL)	12.2 (10.9–13.3)	11.9 (9.6–13.1)
Glycemia (mg/dL)	124 (108–152)	137 (115–170)
Fibrinogen (mg/dL)	353 (297.25–435.75)	395.5 (313.25–472.50)
*Type of fracture*		
Pertrochanteric (%)	1186 (53.6)	57 (58.2)
Femoral neck (%)	804 (36.3)	31 (31.6)
Subtrochanteric (%)	224 (10.1)	10 (10.2)
*CFS group*		
Mild CFS (%)	400 (18.1)	2 (2.0)
Moderate CFS (%)	1396 (63.1)	59 (60.2)
Severe CFS (%)	418 (18.9)	37 (37.8)
*Sex*		
Male (%)	535 (24.2)	38 (38.8)
Female (%)	1679 (75.8)	60 (61.2)
*Tyme to surgery*		
< 24 h to surgery (%)	1343 (60.7)	45 (45.9)
24–48 h to surgery (%)	745 (33.6)	41 (41.8)
> 48 h to surgery (%)	126 (5.7)	12 (12.2)
*Outcomes*		
Fever (%)	47 (2.1)	6 (6.1)
Dyspnea (%)	30 (1.4)	4 (4.1)
Sepsis (%)	4 (0.2)	7 (7.1)
Pneumonia (%)	31 (1.4)	10 (10.2)
*Comorbidities*		
Myocardial infarction (%)	252 (11.4)	21 (21.4)
Heart failure (%)	300 (13.6)	27 (27.6)
COPD (%)	111 (5.0)	17 (17.3)
Diabetes (%)	187 (8.4)	9 (9.2)
Renal failure (%)	58 (2.6)	13 (13.3)
Neoplasm (%)	93 (4.2)	7 (7.1)

Furthermore, in patients with severe CFS, the time to surgery was longer on average (22.5 h vs. 18.8 h in less frail patients, p < 0.001) (Table 2).

**Table 2:** Clinical characteristics of enrolled patients according to clinical frailty scale (CFS); CFS = clinical frailty scale; CCI = Charlson comorbidity Index; Q1–Q3 = interquartile range (25–75th percentile)

Variable	Mild (1–3) CFS (n = 402)	Moderate (4–6) CFS (n = 1455)	Severe (7–9) CFS (n = 455)
Age (years)	78 (70–84.25)	85 (80.0–90.0)	86 (82.0–91.0)
CCI	4 (3.0–4.0)	4 (4.0–5.0)	5 (4.0–6.0)
CFS adjusted	3 (3.0–3.0)	5 (4.0–6.0)	7 (7.0–7.0)
Nottingham mortality (%)	2.37 (1.45–2.37)	2.37 (1.45–3.85)	3.85 (2.37–6.19)
Time to surgery (hours)	18.8 (8.8–27.1)	20.8 (10.2–29.7)	22.5 (11.6–34.6)
*Time to surgery (hours)*			
< 24 h (%)	65.7	60.5	53.4
24–48 h (%)	30.1	33.9	37.8
> 48 h (%)	4.2	5.6	8.8
*Outcomes*			
Death (%)	0.5	4.1	8.1
Fever (%)	2.0	2.2	2.9
Dyspnea (%)	1.0	1.2	2.9
Syncope (%)	1.0	2.7	2.2
Sepsis (%)	0.2	0.5	0.4
Pneumonia (%)	0.7	1.9	2.2
Other infections (%)	0.5	1.2	2.6
Heart attack (%)	0.0	0.2	0.2
Atrial flutter (%)	2.0	5.6	6.8
Stroke (%)	0.0	0.4	1.1
Anemia (%)	3.7	6.6	6.2
Electrolyte imbalance (%)	0.5	0.3	1.1

Increased length of stay (LOS) has been identified as an important factor associated with mortality and post-operative complications. An increase in in-hospital mortality from 2.9% to 5.6% (p = 0.001) was noted in patients with an LOS of more than 7.1 days. These patients experienced a significantly higher incidence of pneumonia (3.3% vs. 0.3%, p < 0.001), sepsis (1.0% vs. 0%, p = 0.001), atrial flutter (7.5% vs. 2.9%, p < 0.001), and anemia (8.8% vs. 3.2%, p < 0.001). Patients with an LOS greater than 7.1 days also had a higher risk profile: those with severe CFS (22.5% in the LOS group > 7.1 days vs. 16.9% in the LOS group < 7.1 days), patients with heart failure (17.2% vs. 14.1%, p = 0.000), and patients with renal failure (4.3% vs. 1.8%, p = 0.000) (Table 3).

**Table 3:** Clinical characteristics of enrolled patients according to length of stay (LOS): LOS = Length of Stay; CCI = Charlson Comorbidity Index; CFS = Clinical Frailty Scale; Hb = Hemoglobin; PT = Prothrombin Time; WBC = White Blood Cell Count; PLT = Platelet Count; COPD = Chronic Obstructive Pulmonary Disease; HIV = Human Immunodeficiency Virus

Variable	LOS < 7.1 days (n = 1156)	LOS > 7.1 days (n = 1156)
Age (years)	84 (78.0–89.0)	85 (79.0–90.0)
CCI	4 (4.0–5.0)	4 (4.0–6.0)
CFS adjusted	4 (4.0–6.0)	5 (4.0–6.0)
Nottingham mortality (%)	2.37 (1.45–3.85)	2.37 (2.37–3.85)
Time to surgery (hours)	19.2 (10.3–27.3)	22.05 (10.4–33.5)
*CFS group*		
Mild (%)	23.1	11.7
Moderate (%)	60.0	65.8
Severe (%)	16.9	22.5
*Laboratory values*		
Hb (g/dL)	12.3 (11.1–13.4)	12.1 (10.5–13.2)
Glycemia (mg/dL)	123 (107.0–151.0)	129 (108.8–157.0)
Blood creatinine (mg/dL)	0.85 (0.67–1.1)	0.825 (0.68–1.11)
Fibrinogen (mg/dL)	345 (289.75–433.0)	364 (307.25–446.0)
PT (seconds)	11.4 (10.95–12.15)	11.5 (11.0–12.5)
WBC (× 10⁹/L)	10.39 (8.46–12.635)	10.2 (8.2125–12.6175)
PLT (× 10⁹/L)	226 (185.0–287.0)	224 (179.25–279.5)
*Sex*		
Male (%)	22.8	26.8
Female (%)	77.2	73.2
*Time to surgery*		
< 24 h (%)	64.7	55.4
24–48 h (%)	32.4	35.6
> 48 h (%)	2.9	9.1
*Outcomes*		
Death (%)	2.9	5.6
Fever (%)	1.6	2.9
Dyspnea (%)	0.9	2.1
Syncope (%)	2.2	2.4
Sepsis (%)	0.0	1.0
Pneumonia (%)	0.3	3.3
Other infections (%)	0.1	2.7
Heart attack (%)	0.1	0.3
Atrial flutter (%)	2.9	7.5
Stroke (%)	0.3	0.7
Anemia (%)	3.2	8.8
Electrolyte imbalance (%)	0.1	1.0
*Comorbidities*		
Myocardial infarction (%)	9.5	14.1
Heart failure (%)	11.1	17.2
COPD (%)	3.6	7.4
Diabetes (%)	8.0	8.9
Liver disease (%)	0.6	1.9
Renal failure (%)	1.8	4.3
Neoplasm (%)	3.4	5.3
Metastasis (%)	0.9	1.2
Lymphoma/leukemia (%)	0.9	1.6
HIV (%)	0.2	0.0
Connective tissue disease (%)	2.2	1.0
Hemiplegia (%)	0.3	0.3

Multivariate logistic regression identified CFS as a significant determinant of in-hospital mortality (p = 0.019). Specifically, patients with moderate frailty (CFS 4–6) had a 4.35-fold increased risk of mortality (OR = 4.351, p = 0.044), while those with severe frailty (CFS 7–9) had a 6.6-fold higher risk (OR = 6.609, p = 0.012), compared to patients with mild frailty (CFS 1–3).

## Discussion

The findings of this study underscore the critical role of frailty in determining clinical outcomes in elderly patients with proximal femur fractures, with the CFS emerging as a key predictor of mortality, prolonged hospital stay, and post-operative complications. Our analysis revealed a progressive increase in mortality risk as frailty severity escalates, with CFS 7–9 patients exhibiting a more than sixfold higher risk of death compared to non-frail individuals. Furthermore, age, male sex, and comorbid burden (Charlson Comorbidity Index) were identified as independent risk factors for adverse outcomes. Another crucial finding was the impact of surgical timing on survival, as delays beyond 48 h significantly increased mortality risk, emphasizing the need for early intervention to mitigate complications. Additionally, prolonged hospital stay (LOS > 7.1 days) was associated with higher rates of infections, cardiovascular events, and overall mortality, highlighting the challenges in post-operative management of frail patients.

This trend was also found in the study by Sagona et al. [15] showing that as the degree of frailty increases, the rate of mortality and re-hospitalization also increases. These results highlight the escalating risk of mortality associated with the degree of frailty, reinforcing the importance of frailty as a prognostic factor in this patient population. Our results are in line with what has been reported in the literature. Narula et al. [10] confirm that 30-day mortality increases from 1.3% (CFS 1–3) to 14.6% (CFS ≥ 7), while one-year mortality reaches 41.7% in patients with CFS ≥ 7. Similarly, Forssten et al. [16] found that frail patients have a fourfold increased risk of 30- and 90-day mortality. However, a study by Ikram et al. [17] suggests that the increase in mortality is not linear beyond a certain degree of frailty, as the risk seems to stabilise for CFS > 5. Furthermore, while our study focused on overall mortality, Forssten et al. [16] analysed cause-specific mortality, showing that frailty is associated with a threefold increase in the risk of cardiovascular and respiratory death and a fivefold increase in multi-organ failure. Finally, in addition to mortality, CFS was found to be an effective predictor of length of hospital stay and the need for post-operative institutionalisation. This association of fragility as a positive predictor of mortality in patients with femur fracture was not found in the study by Miller et al. [18], where over 290 patients who had a fragility femur fracture no statistically significant relationship was found between fragility fracture and mortality. However, in a retrospective study by Hua et al. [19] this strong association between fragility hip fractures and mortality was demonstrated, going to an approximately ninefold increase in mortality risk compared to the general population. Age was also confirmed as an independent risk factor, with each additional year of age increasing the risk of mortality by 6.5% (OR = 1.065, p < 0.001).

The male sex was further identified as a predictor of worse outcomes, with a 1.82-fold higher risk of mortality compared to females (OR = 1.824, p = 0.007). These data were confirmed in the study by Leung et al.[20]; Gender differences play a significant role in post-operative outcomes of femur fractures. Our study showed that men are associated with a 1.82 times higher mortality risk than women (OR = 1.824, p = 0.007). These data are confirmed by Narula et al. [10], who report a significantly higher mortality in men, regardless of age and degree of frailty. Furthermore, Forssten et al. [16] found that male sex, combined with a high CFS score, further increases the probability of death at 30 and 90 days. Furthermore, our study showed that men more frequently have severe comorbidities, such as cardiovascular disease and diabetes, factors that increase the risk of post-operative complications. These data are confirmed by Wehren et al.[21], who report a higher incidence of hypertension and metabolic diseases in men with hip fracture. A key aspect that emerges from the literature is that men are less likely to receive adequate rehabilitation care. Our study shows that men are more frequently discharged in poor condition, increasing the risk of hospital readmission. Wehren et al. [21] support this observation, pointing out that men are less likely to be transferred to rehabilitation facilities than women, probably due to differences in social support and post-operative clinical management. However, some studies suggest that biological differences between the sexes could explain part of the mortality gap. Wehren et al. [21], report that oestrogen may have a protective effect on women, improving cardiovascular and bone health, and reducing the risk of post-fracture complications. Furthermore, differences in inflammatory and immune mechanisms between men and women could promote better post-operative recovery in women. These data suggest that more careful post-operative management of men with femur fracture could help reduce their higher mortality risk. Improving access to rehabilitation and monitoring of comorbidities could be key to reducing the survival gap between the sexes.

Concerning comorbidities, the Charlson Comorbidity Index (CCI), which evaluates the overall burden of pre-existing illnesses, was strongly linked to mortality rates.

These results are consistent with those of Miettinen et al. [22], who showed that patients with CCI ≥ 4 have a 3.1–8.5 times higher risk of death than those with lower CCI. Other studies in the literature corroborate these data, Atthakomol et al. [23] showed that specific comorbidities such as: chronic obstructive lung disorders, Alzheimer’s disease, dementia, and active malignancy or low hemoglobin levels at emergency department admission are associated with an increased risk of mortality. Similarly Abeygunasekara et al. [24] in their study demonstrated a close correlation between comorbidity burden assessed by the Charlson comorbidity index and mortality in patients with fragility femur fractures. These results highlight the substantial influence of comorbid conditions on clinical outcomes in femoral fracture patients, particularly in those with higher levels of frailty. These patients are more likely to have multiple comorbidities, which exacerbate the severity of their condition and hinder their recovery after surgery. Furthermore, the role of the CCI in surgical risk stratification was also highlighted by Xing et al. [25], who developed an adjusted CCI including operative delay and age, which improved the prediction of one-year mortality. However, while our study mainly focuses on mortality, Miettinen et al. [22] observed that a high CCI is also associated with a significant increase in post-operative complications, such as cardiovascular events and infections. These data might suggest that the CCI should not only be used to predict mortality, but also to optimize the perioperative management of frail patients.

The analysis revealed no statistically significant correlation between fracture type and mortality. These results are in line with other works in the literature. A study by Walter et al. [26] reported fracture location as a remarkable factor, with a one-year mortality rate of 26.8% for femoral neck or head fractures and 28.2% for fractures involving the trochanteric region. This suggests that while the type of fracture may play a role in clinical outcomes, it is the patient’s underlying frailty and comorbidities that primarily drive the prognosis.

A critical factor identified in this analysis is the time to surgical intervention, which was confirmed as a strong predictor of mortality. This concept is reported by many articles in the literature. According to Carretta et al. [27], surgery after 48 h in proximal femoral fractures increases the risk of mortality at 30 days. Greve et al. [28] demonstrated this association only for patients with multiple comorbidities who have an ASA score of 3 or 4, however in patients without comorbidities this association between mortality and delay in treatment has not been demonstrated. However, Farrow et al. [29] did not show differences in mortality at 30 days and 60 days in patients operated for proximal femoral fracture both within 36 h and after 36 h. Klestil et al. [30] demonstrated a 20% mortality reduction in patients operated within 48 h. Liu et al. [31] also confirm that patients operated on after 48 h have a significantly higher risk of postoperative complications, such as infection and thromboembolism. However, some studies have shown that surgical delay may not influence mortality in less frail patients. Joseph et al. [32] found no significant differences in 1-year mortality between patients operated before or after 48 h, suggesting that other factors, such as comorbidities, may play a key role. Furthermore, recent studies have highlighted possible risks associated with ultra-early surgery (< 12 h). Liu et al. [31] noted that although it reduces medical complications, ultra-early surgery increases the risk of bleeding. Finally, the impact of delay varies depending on the type of hospital facility. Joseph et al. [32] found that patients treated in community hospitals experience greater delays than those operated in Level 1 trauma centres, which could influence clinical outcomes.

Our results highlight the importance of timely surgical intervention, particularly in frail patients, to minimize complications and mortality risk. While reducing surgical delays is essential, a comprehensive preoperative assessment and appropriate hospital management are also crucial for optimizing clinical outcomes. Prolonged waiting times may exacerbate physiological deterioration, increasing susceptibility to infections and adverse events.

The length of stay (LOS) was also a key factor associated with mortality and postoperative complications. This is in line with the study by Ek et al. [33], which showed that a LOS beyond 12 days significantly increased mortality at 4 months, with the effects being more pronounced in patients with pre-existing comorbidities. These results underline the fact that frailty, by prolonging recovery and complicating the management of comorbidities, is a key factor in the worsening of clinical outcomes and increased mortality in femoral fracture patients. However, some studies suggest that the relationship between LOS and mortality is not linear, but follows a U- or J-shaped pattern. Ek et al. [33] observed that both a very short (2–4 days) and a very long (> 24 days) LOS are associated with increased mortality, indicating that the patient’s clinical context plays a key role. Another aspect to consider is that early discharge may not always be beneficial. Ek et al. [33] find that patients discharged early from nursing homes have a higher mortality, probably due to lack of adequate care after discharge. In contrast, in patients discharged home, a shorter LOS appears to be associated with a lower risk of mortality, probably due to increased mobilisation and faster functional recovery. These data underline the importance of personalised management of the length of hospital stay, balancing the risks of a prolonged stay with those of an early discharge.

The analysis of deceased patients reinforced the crucial impact of frailty and clinical management on mortality outcomes. Prolonged hospital stays were more frequent among deceased patients, who mainly succumbed to sepsis, pneumonia, and heart failure. Elevated blood glucose and fibrinogen levels suggested an inflammatory and hypercoagulable state, increasing the risk of thromboembolic and infectious complications. This is in line with the study by Raichandani et al. [34], which showed that lung infections (9%) and cardiac complications (5%) are among the main factors in post-fracture mortality.

**Fig. 1:**
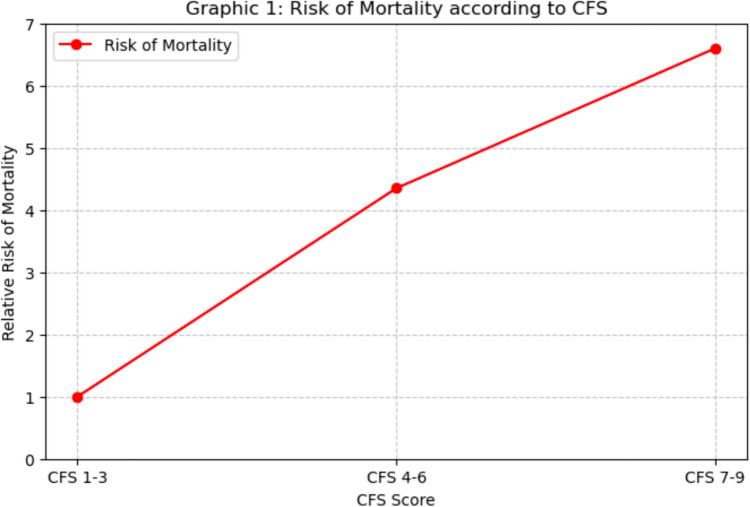
Risk of mortality according to CFS (Clinical frailty scale)

The analysis of all these parameters shows that as the patient’s frailty status (CFS score) increases, there is an inherent increase in the relative risk of mortality Fig. 1.

**Fig. 2:**
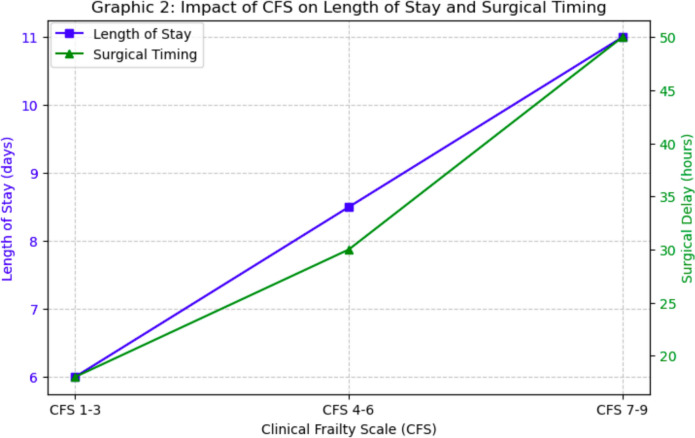
Impact of CFS on length of stay and surgical timing (CFS: Clinical frailty scale)

This risk is also indirectly related, since increased frailty causes more complex pre-operative management (and thus increased delay in surgery) but also more complex post-operative management (and thus increased LOS) (Fig. 2).

## Conclusions

This study reinforces the strong relationship between frailty and outcomes in patients with proximal femur fractures. The Clinical Frailty Scale (CFS) has proven to be a crucial predictor of mortality, complications, and prolonged hospitalization, underscoring the need for systematic frailty assessment in orthopedic practice. Frailty influences not only survival but also functional recovery, increasing the likelihood of post-operative institutionalization and limiting rehabilitation potential. Another significant finding is the impact of surgical timing. Delays beyond 48 h were associated with a marked increase in mortality, likely due to prolonged immobilization, a higher risk of thromboembolic events, and worsening systemic decompensation. These findings highlight the importance of optimizing hospital workflows to prioritize early surgery, particularly in frail patients who are more vulnerable to adverse events. Hospital length of stay (LOS) also plays a critical role. While prolonged LOS is associated with a higher incidence of complications such as infections and cardiac events, excessively early discharge could compromise recovery. Identifying the optimal balance between surgical timing, hospital stay, and rehabilitation strategies remains a key challenge in orthopedic management.

Future studies should explore personalized surgical approaches based on frailty status, evaluating the impact of different fixation techniques and early mobilization protocols. Additionally, integrating frailty indices with orthopedic scoring systems could refine risk stratification and improve decision-making. A multidisciplinary approach that combines orthopedic expertise with geriatric care and rehabilitation planning will be essential in improving outcomes for this high-risk population.

## Data Availability

All the data we analyzed and tables we compiled are available for any clarification.
